# ACL microtrauma: healing through nutrition, modified sports training, and increased recovery time

**DOI:** 10.1186/s40634-022-00561-0

**Published:** 2022-12-14

**Authors:** J. Nyland, B. Pyle, R. Krupp, G. Kittle, J. Richards, J. Brey

**Affiliations:** 1 Norton Orthopedic Institute, 9880 Angies Way, Louisville, KY 40241 USA; 2 MSAT Program, Spalding University, 901 South Third St, Louisville, KY USA; 3 Department of Orthopaedic Surgery, University of Louisville, Louisville, KY USA

## Abstract

**Purpose:**

Sports injuries among youth and adolescent athletes are a growing concern, particularly at the knee. Based on our current understanding of microtrauma and anterior cruciate ligament (ACL) healing characteristics, this clinical commentary describes a comprehensive plan to better manage ACL microtrauma and mitigate the likelihood of progression to a non-contact macrotraumatic ACL rupture.

**Methods:**

Medical literature related to non-contact ACL injuries among youth and adolescent athletes, collagen and ACL extracellular matrix metabolism, ACL microtrauma and sudden failure, and concerns related to current sports training were reviewed and synthesized into a comprehensive intervention plan.

**Results:**

With consideration for biopsychosocial model health factors, proper nutrition and modified sports training with increased recovery time, a comprehensive primary ACL injury prevention plan is described for the purpose of better managing ACL microtrauma, thereby reducing the incidence of non-contact macrotraumatic ACL rupture among youth and adolescent athletes.

**Conclusion:**

Preventing non-contact ACL injuries may require greater consideration for reducing accumulated ACL microtrauma. Proper nutrition including glycine-rich collagen peptides, or gelatin-vitamin C supplementation in combination with healthy sleep, and adjusted sports training periodization with increased recovery time may improve ACL extracellular matrix collagen deposition homeostasis, decreasing sudden non-contact ACL rupture incidence likelihood in youth and adolescent athletes. Successful implementation will require compliance from athletes, parents, coaches, the sports medicine healthcare team, and event organizers. Studies are needed to confirm the efficacy of these concepts.

**Level of evidence:**

V

## Introduction

As participation in youth and adolescent sports continues to increase, a direct impact on injury rates, medical costs, family burden, and time away from sport is observed [61]. Despite an abundance of well-designed exercise training approaches to address modifiable primary anterior cruciate ligament (ACL) injury prevention, injuries continue to occur at a high frequency among adolescent and youth sport athletes [61, 63, 82]. The athlete remembers the instant “pop” of sudden ACL failure. Unfortunately, little if any consideration is given to the less “eventful” sports training microtrauma accumulation that preceded it [22, 56, 104, 113]. We still don’t understand why an athlete can perform a single leg jump landing or running direction change pivot maneuver over and over again without injury, yet suddenly rupture their ACL when performing the same maneuver one more time [22, 56]. This clinical commentary reviewed medical literature related to non-contact ACL injuries among youth and adolescent athletes, collagen and ACL extracellular matrix metabolism, ACL microtrauma and sudden failure, and concerns related to current sports training. This information was then synthesized into a comprehensive intervention plan for the purpose of better managing ACL microtrauma, thereby reducing the incidence of non-contact macrotraumatic ACL rupture.

## Non-contact ACL injuries among youth and adolescent athletes

**Fig. 1:**
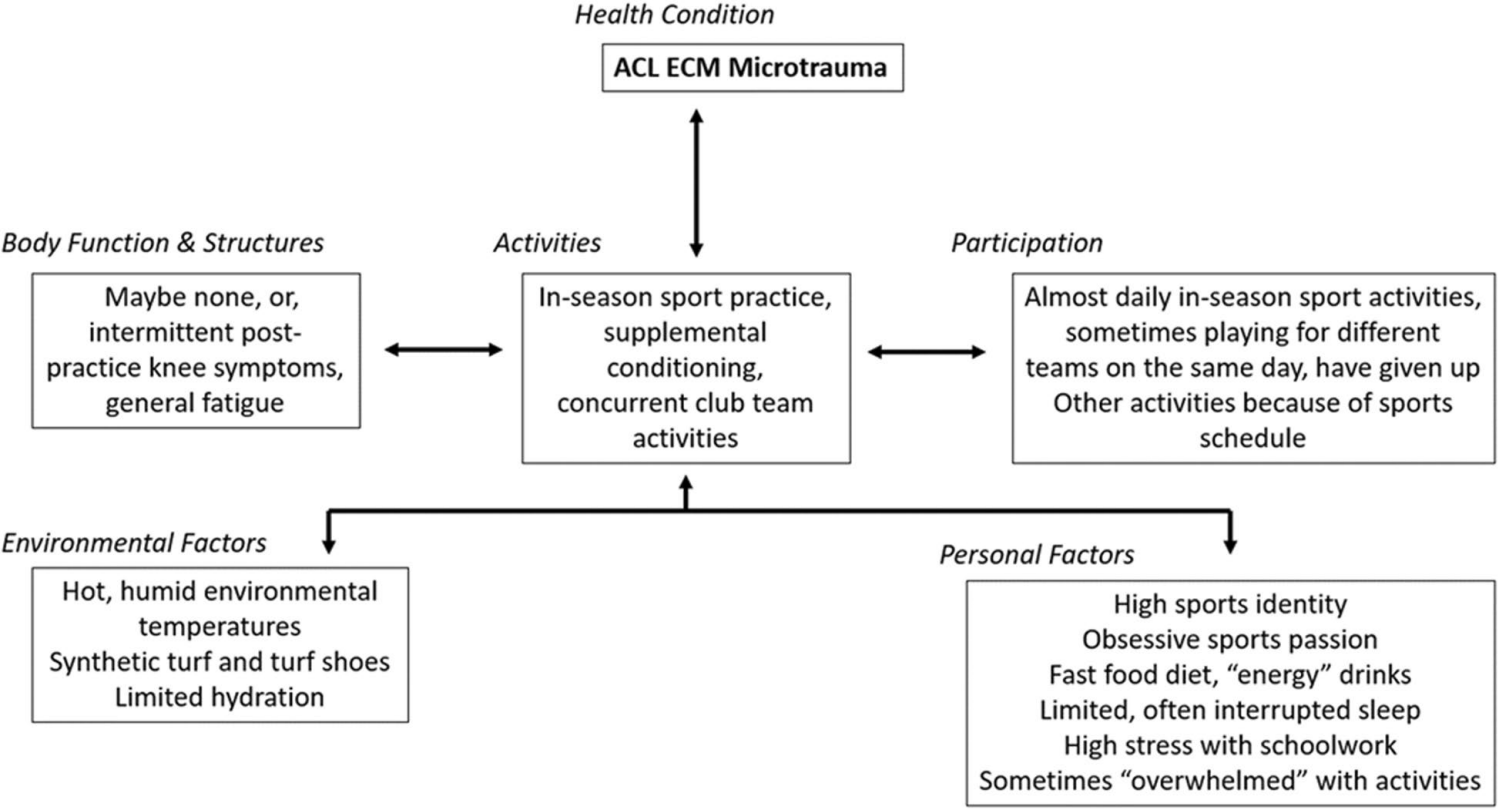
ACL extracellular matrix microtrauma example within the International Classification of Functioning, Disability and Health (ICF) framework [107]. Many youth and adolescent sport athletes display variable combined factors that lead to ACL microtrauma accumulation

Knee ligament disease is a major healthcare concern [12, 58] and locomotory system injuries from sports are public health problems that contribute to a worldwide disability burden [61, 81]. Degenerative knee joint changes from acute ligament injuries represent a large portion of sports rehabilitation practice [16] and the best method for treating them is far from resolved [65, 67]. Among active people, ACL injury has an incidence as high as 1 in 3000 [54] and nearly 75% of all ACL injuries occur without contact, even in high collision sports [13, 82]. Pediatric ACL tears represent most of these injuries, particularly among young female soccer and basketball athletes and in many, surgery is complicated by potential epiphyseal plate injuries [37]. Non-contact ACL injuries are occurring concurrently with increased organized sports and recreational activity involvement by girls and young women who are predisposed to the added influence of monthly menstrual cycle hormonal effects, and more frequent dynamic malalignment during single lower limb loading [58, 84]. The best current example of gender equity in sports may be overuse injury frequency. Since ACL injuries among youth and adolescent athletes may involve varying combinations of body function/structure, activity/participation, or environmental/personal factors, use of a biopsychosocial model such as the International Classification of Functioning, Disability, and Health [103, 107] may provide the sports healthcare team with a helpful method to comprehensively consider which factors might be most relevant for each individual athlete. The injury potential for accumulated microtrauma ACL injury in youth and adolescent athletes is currently unknown [22]. However, a growing body of evidence suggests that given the total sports training volume performed by most youth and adolescent athletes today, accumulated ACL microtrauma should be considered as an extrinsic, modifiable risk factor of high importance (Fig. 1).

## Collagen and ACL extracellular matrix metabolism

The extracellular matrix (ECM) is a non-cellular, three-dimensional macromolecular scaffold consisting of collagen, enzymes, and glycoproteins that provide biochemical and biomechanical cues crucial to ligament morphogenesis, differentiation and tensional homeostasis [1, 14, 92]. Healthy ligaments are primarily comprised of type I and II collagen, along with glycosaminoglycans (GAGs) that surround and align them into a hierarchy of organized fibrils, fascicles, and fibers orientated along the ACL axis increasing its strength and resilience [45, 81]. Ligaments are 90% type I collagen, but after injury, higher type III collagen volume initially exists [62, 81]. To better withstand loads, the ACL responds to ECM mechanical and chemical environment changes through biomechanical adaptations [62, 111]. Increased ECM degradation or “turnover” is associated with higher strain levels [77]. With immobilization, collagen synthesis decreases, anerobic and aerobic tissue metabolism is reduced, and ligament strength decreases. With restored mobility, ECM biomechanical properties begin to return to normal, however, ligament insertions (entheses) take longer to recover than other regions [106, 112].

**Fig. 2:**
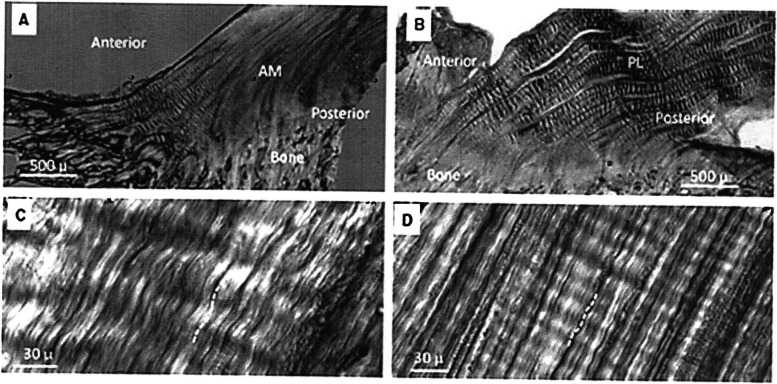
**A** Crimping in the anteromedial (AM) ACL bundle; **B** Crimping in the posterolateral (PL) bundle; Coarse crimps are visible in the anterior AM bundle and throughout the PL bundle; **C** High magnification view of course crimping in the AM bundle; **D** High magnification view of fine crimping in the posterior AM bundle. Figure used with permission [112]

Collagen crimp provides a functional buffer to immediate longitudinal ACL elongation under tensile loads with the nonlinear stress-strain curve “toe region” representing the gradual extension of these crimps [73, 74] (Fig. 2). As the “toe region” transitions into the linear stress-strain curve region, the crimps become completely extended, and resistance is provided by collagen triple helix fibers with the cross linkages stretching between these helices [111]. Toe region behavior is important because ACL strains during routine activities of daily living, sports training and rehabilitation primarily occur within this tissue loading zone. Crimp pattern morphological variations provide insight into ACL bundle function. In an ovine model, Zhao et al. [111] reported that the anteromedial ACL bundle functions more during weightbearing stance stabilizing the knee by preventing anterior tibial translation [6, 111], while the posterolateral ACL bundle functions more near maximum extension and flexion to control internal-external tibial rotation [111, 112]. Less crimping results in earlier stiffness (smaller toe region) as the ACL is stretched.

## ACL microtrauma and sudden failure

Microtrauma represents small amounts of structural ECM damage from repeated sub-failure loading that accumulates when its occurrence frequency outpaces natural repair [55, 113]. The magnitude of microtraumatic ACL damage that occurs from routine sports training is poorly understood [22, 56]. Microtrauma-induced vasculature damage may increase intraligamentous or intrasynovial pressure, reducing blood flow, increasing tissue hypoxia, and increasing collagen degeneration [32, 46]. To minimize necrosis during naturally occurring periods of prolonged ischemia, ligaments possess lower metabolic activity rates than bone, cartilage, or muscle, and are more resistant to anerobic conditions during high force transmission [81]. Compared to other tissues, ACL healing occurs more slowly in relationship to applied movements and stresses [45, 72]. High anerobic capacity is an important healthy ligament trait, however, poor vascularity may further delay microtrauma healing [87], with ligament insertion enthesis mechanical properties taking longer to recover from microtrauma than adjacent regions [22, 105]. Femoral ACL enthesis failure may represent accumulated collagen fibril and fiber fatigue damage from accumulated microtrauma that has not had sufficient healing time [22].

**Fig. 3:**
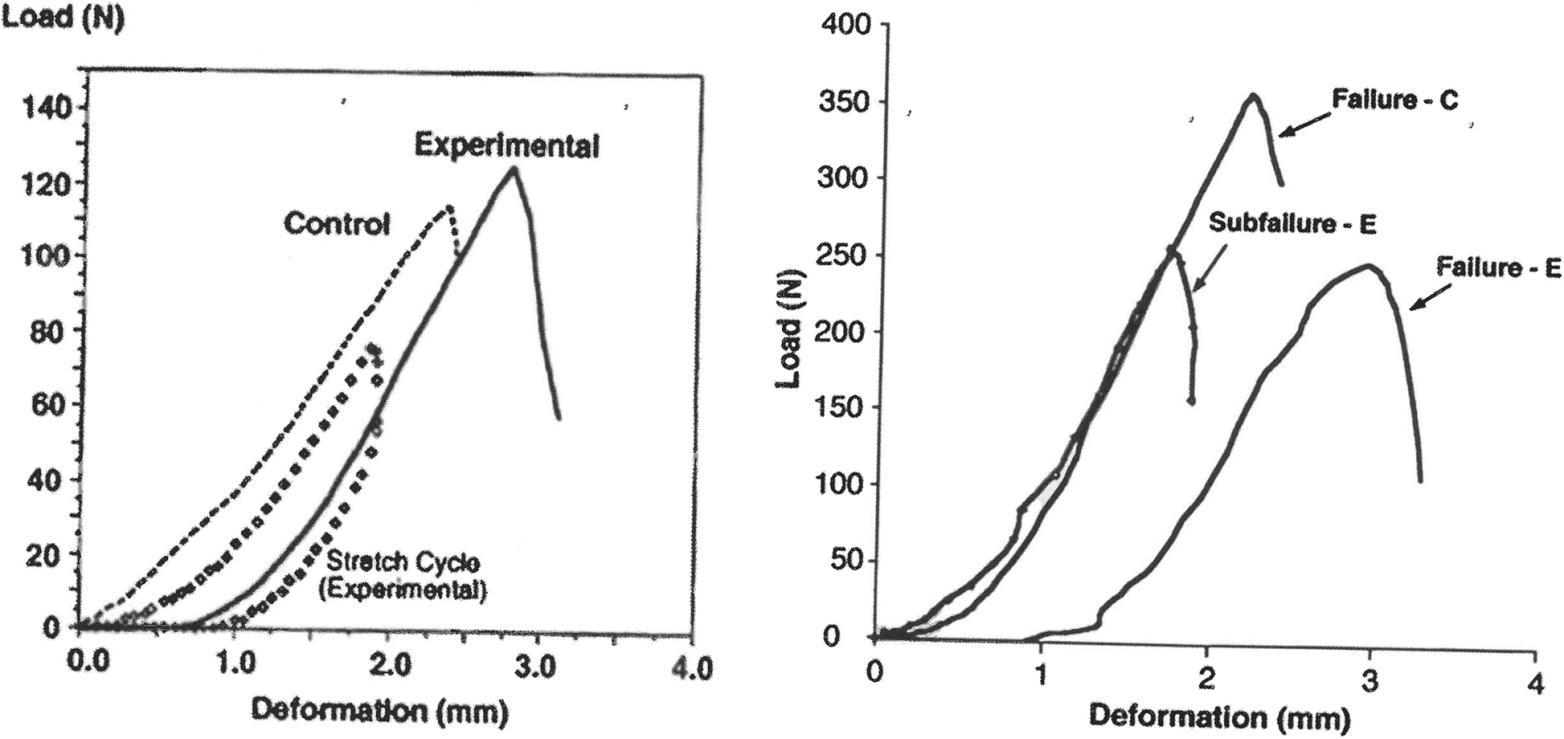
**A** Load deformation curves until failure of a typical pair of experimental and control ligaments with the load and unloading curves of the experimental ligament (hysteresis) for the 80% subfailure stretch [74]; and **B** Representative load deformation curves for experimental (E) and control (C) ligaments. Also shown is the load deformation curve of the 80% subfailure stretch of the experimental ligament (subfailure – E) [73]. Findings confirm impairment in both submaximal hysteresis patterns and deformation under failure loads for the partially injured ACL. Figures used with permission [73, 74]

When the ACL microtrauma rate exceeds the biological repair rate, injury or failure may occur under normal loading conditions [22, 80] as accumulated microtrauma decreases ligament modulus of elasticity leading to sudden fatigue failure [95]. There can be considerable collagen fiber disruption and disorganization in the human ACL at ultimate tensile failure without any visual macroscopic tearing evidence [46, 66]. Collagen triple helix mechanical unfolding may be the key mechanism responsible for the generation of fascicle-level creep strain that leads to sudden ACL fatigue rupture under cyclic loading conditions [113]. In addition to concerns about sudden fatigue failure following microtrauma, the ACL “toe region” may be elongated [73, 74], potentially yielding greater laxity during routine activities [56]. Using an in vivo rabbit model, Panjabi et al. [73] found that after sub-failure ACL injury (80% of the contralateral control ACL), the ultimate load, deformation, and energy absorbed at failure did not change under high speed loading. However, the load deformation curve displayed a “toe region” hysteresis curve that was only 30% of the energy to failure of the control ligament during the sub-failure stretch cycle, suggesting damage to longitudinal collagen fibers and crosslinks [73] (Fig. 3). In a follow-up study, Panjabi and Courtney [72] found that the same sub-failure injury also increased ACL ultimate deformation. Accumulated microtrauma reduces ACL mechanical properties [46, 80] and lengthens the force-displacement curve “toe region” resulting in increased laxity [74, 80]. Both accumulated microscopic ECM degradation and outright type I collagen destruction impairs ACL mechanical function. Stress-strain curve “toe region” shape, tangent modulus, and ECM tensile strength are directly dependent on fibrillary and molecular level collagen crosslinks [38]. The accumulation of damage over the course of fatigue failure reduces a ligament’s modulus of elasticity [95], increasing its susceptibility to sudden failure. This may be especially true for girls and young women, given their smaller ACL diameter and volume and decreased modulus of elasticity [13, 20, 21]. Depending upon load magnitude, human ACL fatigue life can be as few as sixty severe loading cycles, with a larger force and a smaller cross-sectional area being predictive of greater sudden failure risk [56]. Studies are needed to better determine how recovery time between ACL loading cycles might improve its resistance to fatigue failure [56]. Longer or re-proportioned rest intervals between sports training loading cycles may help decrease cumulative ACL strain, thereby increasing fatigue life [79]. Therefore, thoughtful management of the number of knee pivot-type landing maneuvers that an athlete performs over a short time period may be helpful [56, 71].

Sudden running deceleration, directional changes, or single limb loading with valgus knee stress are known to create large, potentially injurious ACL loads [4, 29, 36, 110]. Healthy entheses dissipate loading stresses away from the ACL insertions, however, they are highly susceptible to degeneration from overuse [11, 33], also possessing high surgical failure rates [57]. Given their lesser vascular density and smaller diameter blood vessels, enthesis remodeling occurs more slowly than other ACL regions [5, 96], leading to greater potential for knee osteoarthritis and severe disability [15]. The femoral ACL enthesis has a 3.9 times more acute ACL attachment angle, 43% greater calcified fibrocartilage area, and 226% greater uncalcified cartilage depth than the tibial enthesis [9]. These characteristics in combination with hip internal-external rotational alignment during sudden single leg jump landings or running directional change movements may influence both accumulated microtrauma and sudden ACL failure [10, 104]. As the hip approaches terminal internal rotation, peak ACL strain suddenly increases from direct femoral neck and acetabular rim contact [10, 104]. Non-uniform ACL stresses from accumulated regional microtrauma, neurally-mediated substance P release from primary afferents, and prolonged exercise-induced hyperthermia can also create the central core ACL ECM degeneration that precedes ACL rupture [85, 91]. Although a healthy biological response to loading modifies microtrauma accumulation, high intensity, frequency or total volume sports training may contribute to the loss of natural repair homeostasis increasing non-contact sudden ACL rupture risk.

## Concerns about sports training practices

We have a poor understanding of how biomechanical overload timing and severity from sports training influences the in vivo proteolytic activity that may drive ACL microtrauma beyond the threshold for natural repair homeostasis [19, 113]. High frequency, intensity, or total volume sport training may create situations where youth and adolescent athletes are at greater risk for compromising any of a number of developmental processes through chronic overtraining and hormonal dysregulation [37, 58, 61, 68]. Through mechanotransduction, ACL cells sense mechanical environment changes and respond by modulating biochemical mediators [12]. The nature and acuity of this healing response can prompt either an anabolic, homeostatic, or catabolic state, in which ECM production and structural properties are respectively either increased, maintained, or reduced. Increased ECM collagen production and incorporation occurs within hours of loading [53, 86] with circadian regulation of collagen synthesis, cellular export, and collagen degradation attempting to maintain or restore tissue homeostasis [19]. Excessive sports training intensity, frequency, or total volume, however, may upset this balance.

Periodization principles were developed to improve neuromuscular or cardiopulmonary system endurance, strength or power [43, 48, 52]. These principles, however, do not adequately address individualized youth or adolescent athlete growth, recovery and remodeling variability, cognitive development, epiphyseal region maturation, emotional development or hormonal changes [43, 47, 52]. The homeostasis recovery requirements of lower metabolic rate ligaments and tendons are likewise not considered. Perhaps sports training periodization can be adapted to better promote ACL ECM homeostatic balance, thereby enhancing microtrauma recovery, healing, and remodeling. Sport training intensity, frequency and total volume have been widely studied for physiological and psychological performance purposes. In contrast, how best to optimize ACL ECM microtrauma recovery and maintain homeostasis in harmony with improved sport performance is poorly understood.

## Developing a comprehensive primary non-contact ACL injury prevention plan

Although not all injuries can be prevented, it appears that the youth and adolescent sport culture is falling short in minimizing both traumatic and overuse injuries. Parents, coaches, the sports healthcare team, and event organizers are all culpable [61]. At least 50% of the injuries sustained by young athletes result from overuse from intrinsic (some non-modifiable) or extrinsic modifiable factors [61]. The plan we propose focuses on key extrinsic or modifiable factors that to date have been largely ignored. For today’s youth or adolescent athlete, sport training, conditioning, or competition often consumes most days of the week [61, 68]. With these considerations and the fact that the knee is the primary overuse injury location [82], we should take advantage of every opportunity to improve primary non-contact ACL injury prevention. To better understand how these injuries occur, it is important to not just consider risk factors and injury mechanisms, but also protective factors and “mechanisms of no injury” [60]. Early sport specialization or “professionalism”, high sport training intensity, frequency, total volume, excessive focus on winning or early talent development, prior injury, peripheral and central fatigue, poor neuromuscular control/dynamic joint stability, psychosocial stresses, extended sports seasons playing for multiple teams, and limited recovery time may refract injury prevention efforts [44, 61, 69, 70, 89, 100]. Many adolescent and youth athletes could also benefit from consultation with a nutritionist who promotes a “food first approach” to prevent and treat injuries [28, 61]. Proper nutrition and appropriate exercise mode and dosage may improve ACL ECM integrity [50]. Healthy sleep and nutrition are essential connective tissue recovery and healing factors [34]. The rise of technology has led modern man to assume more sedentary lifestyles compared to our ancestors, with greater access to, and volume of daily and nightly computer and handheld device use, e-gaming, and the ingestion of calorie dense/nutrient level low “energy drinks or foods” [34, 68]. Factors such as these may heighten sympathetic nervous system activation increasing the plasma cortisol levels that compromise the capacity for the parasympathetic nervous system to effect microtrauma recovery, healing, and remodeling [2, 34, 68]. Should athletes decide to intermittently use non-steroidal medications to control knee pain or inflammation [97, 101], they may further impair the healing process [23, 24, 51]. Zitnay et al. [113] confirmed that molecular level collagen damage from microtrauma initiated the ligament fatigue process that ultimately progresses to complete failure during repetitious submaximal cyclic loading.

We propose an innovative sport training approach that periodizes dedicated sport skill/strategy training with more ACL intense physical conditioning days. Since ACL health is highly dependent on maintaining a collagen-rich ECM with strong cross-linking [28], in addition to ensuring adequate total energy intake and nutritional requirements, the addition of certain supplements may accelerate microtraumatically damaged ACL healing [17, 98]. Bovine hydrolyzed collagen or collagen peptides are short chain amino acids with type I and III collagen that can be absorbed more rapidly into the circulatory system than collagen obtained from gelatin or other dietary sources [8, 88]. Amino acids enriched in collagen (proline, hydroyproline, and hydroxylysine) combined with vitamin C are known to enhance collagen synthesis [8, 75, 88]. Although ligament ECM consists primarily of type I collagen, type III collagen is advantageous during early healing because it more rapidly creates the cross-links that stabilize the damaged ACL loading curve “toe region”. Collagen synthesis and linkages are further enhanced with daily dietary requirements of zinc, sulfur containing amino acids, and beta-carotene [28, 99] (Table 1).

**Table 1:** In addition to 1 mg/kg bodyweight/day of vitamin C for collagen cross-link development, overall protein consumption of 2.3 g/kg bodyweight/day for tissue repair; vitamin D and calcium for bone and enthesis health, and vitamin A for anti-inflammatory effects, athletes should have a healthy diet with sufficient hydration and electrolyte ingestion

Supplement Focus	Diversify diet to include some comfort foods	Pre-Intense practice CHO meal	30–60 minutes before training, athletes consume approximately 15 g of vitamin C-enriched gelatin or the collagen peptide equivalent (both proportional to BW) to increase post-exercise collagen synthesis	Pre-Intense practice CHO meal	30–60 minutes before training, athletes consume approximately 15 g of vitamin C-enriched gelatin or the collagen peptide equivalent (both proportional to BW) to increase post-exercise collagen synthesis	Pre-game day CHO meal	Pre-game CHO meal
Modality	Thermal modalities, massage, stretching/soft tissue mobilization	Cryotherapy or contrasting thermotherapy post-practice	Cryotherapy or contrasting thermotherapy post-practice	Cryotherapy or contrasting thermotherapy post-practice	Cryotherapy or contrasting thermotherapy post-practice	Thermal modalities, massage, stretching/soft tissue mobilization	Post-game cryotherapy
Day of the Week	Sunday	Monday	Tuesday	Wednesday	Thursday	Friday	Saturday

*CHO* complex carbohydrate

During microtrauma healing and remodeling, collagen is primarily added to the ACL periphery as it adapts to loading [8] with greater deposition occurring after relatively short duration acute exercise bouts [26, 39, 42]. Combining ACL loading with appropriate nutritional support is essential [8, 28, 88, 99]. In a randomized, double-blind crossover clinical trial of 8 healthy men (27 ± 6 years of age), ingestion of 15 g of gelatin 1 hour before 6 minutes of jump roping doubled the collagen synthesis rate of engineered human ligaments within 1 hour post-exercise, increasing collagen density and improving tissue mechanics compared to placebo, or low-gelatin groups [88]. In a study of 12 healthy men (22 ± 2.5 years of age), the blood serum growth hormone levels in blood drawn 15 minutes after an acute exercise bout were 7 times greater than serum levels at rest, and engineered ligaments displayed increased collagen content with enhanced tensile loading strength [102]. Baar [8] recommended integrating approximately 10 minutes of training targeting the injury prone ligament, to be performed either 6 hours before or after any other sport training. Thirty to 60 minutes before training, athletes should consume approximately 15 g of gelatin in either liquid or gel form for rapid absorption (proportional to bodyweight) [88]. An intermittent exercise program consisting of 10 min of acute training followed by 6 hours of rest over 5 days has been found to produce more collagen in engineered human ligaments than more continuous training [76]. Having a daily protein intake of 2.3 g/kg bodyweight/day can also facilitate tissue repair [28, 99]. Vitamin D and calcium supplementation improves ligament enthesis and bone health and strength [99]. Omega-3 polyunsaturated fatty acids can provide natural anti-inflammatory effects and vitamin C intake of 1 mg/kg bodyweight/day is essential for collagen cross-link development [28]. Relatively short duration, more acute exercise training bouts also increases the expression of lysl oxidase the primary enzyme involved in collagen synthesis and cross-linking [40], increasing collagen synthesis [31], creating a denser, stiffer, and stronger ECM [30]. In a study of patients ≥49 years of age with mild-to-moderate severity knee osteoarthritis, McAlindon et al. [59] found that the daily consumption of 10 g of collagen hydrolysate improved medial and lateral tibial hyaline cartilage health in patients with mild knee osteoarthritis. In agreement with this finding, a 24-week randomized clinical trial in college age varsity sport and club team athletes with activity-related knee pain showed that collagen hydrolate significantly decreased pain levels [27]. Vitamin C is an essential collagen synthesis component for activating the lysyl oxidase, prolyl, and lysyl hydroxylasese enzymes that increase cross-linking. Accelerated collagen synthesis occurs as early as 4-hours post-exercise enhancing ACL ECM tensile strength through greater deposition and increased cross-linking [88]. These results are similar to what occurs to bone in vivo with very few loading events followed by 6–8 h of rest resulting in the greatest bone mineral deposition [18] (Table 2).

**Table 2:** Example of a modified in-season weekly training progression for high ACL injury risk sports like soccer, basketball, team handball, lacrosse

Focus	Recovery activities (waste removal, nutrient delivery); repetitive low load knee ROM, walking, swimming, bike	Standard practice but with light cutting, running direction changes, jump stops	Shorter duration practice with more intense cutting, running, directional changes and jump stops	Standard practice but with light cutting, running direction changes, jump stops	Shorter duration practice with more intense cutting, running, directional changes and jump stops	Pre-game planning, walk through (waste removal, nutrient delivery)	Competition
Hours/Activity	Off; Active Rest	Moderate intensity sport practice, 2 hr. duration followed by recovery activities	Intense cutting for 10 min in a 1 hr. duration practice; 6–8 hr. rest, then repeat	Moderate intensity sport practice, 2 hr. duration followed by recovery activities	Intense cutting for 10 min in a 1 hr. duration practice; 6–8 hr. rest, then repeat	Low intensity 2 hr. duration	Game; Post-Game recovery activities
Day of the Week	Sunday	Monday	Tuesday	Wednesday	Thursday	Friday	Saturday

In reviewing the entire training and competition schedule, the coaching and sports medicine staffs would develop a similar plan of best fit that follows the recommended progression

Even though sport training frequency, intensity, and total volume influences ligament cellular process regulation, the precise effects of different intervals remains unknown [22, 56, 113]. The current literature suggests that sport training periodization [43, 58] should be modified to better prevent ACL microtrauma accumulation among youth and adolescent athletes. This may be particularly helpful in that youth and adolescent athletes possess more robust neurovascular system mediated healing capability than adults [3, 83, 109]. Since the human genetic code evolved from migratory, low intensity and long duration aerobic metabolism [49, 64, 90], low demand aerobic activities such as submaximal effort cycling or walking, and soft tissue mobilization may further assist circulatory system nutrient delivery to the healing ACL ECM, thereby increasing collagen deposition and metabolic waste removal [94]. An additional aerobic activity benefit is that it may simultaneously improve overall youth or adolescent athlete moods [68]. Growth hormone secretion is increased during sleep, when energy demands are low and the secretion of stress-activated hormones such as cortisol and adrenaline is inhibited [34]. Decreased computer or phone screen time prior to sleeping may help decrease sympathetic nervous system arousal and associated hormonal responses re-balancing parasympathetic nervous system function [homeostasis] [61, 68]. Embedded within basal aerobic metabolism resides the greatest tissue healing capacity through circulatory system nutrient delivery in the presence of repetitious low load active knee movements that facilitate nutrient transfer across the synovial joint membrane [68, 83, 93, 108]. With more balanced sport training recovery, cortisol stress hormone levels should decrease, and exercise-induced blood and plasma mediated healing factor delivery should increase [102]. Cryotherapy alone or in contrast cycles with thermotherapy may increase circulatory system responses by facilitating the reflex vasoconstriction that enhances venous blood extravasation followed by the vasodilation that delivers nutrient-rich blood and plasma-mediated healing factors to the recovering tissues [35, 78]. This may also help alleviate the elevated ligament temperatures that promote ACL ECM collagen core degeneration [85, 91]. Modified sport training periodization should be implemented in harmony with existing neuromuscular control and dynamic joint stability training activities that stimulate the cognitive engagement and motivation needed for positive sensorimotor cortex remodeling [7, 25, 41, 48].

## Conclusion

Preventing non-contact ACL injuries may require greater consideration for reducing accumulated ACL microtrauma. Proper nutrition including glycine-rich collagen peptides, or gelatin-vitamin C supplementation in combination with healthy sleep, and adjusted sports training periodization with increased recovery time may improve ACL ECM collagen deposition homeostasis, decreasing the sudden non-contact ACL rupture incidence likelihood in youth and adolescent athletes. Successful implementation will require compliance from athletes, parents, coaches, the sports medicine healthcare team, and event organizers. Studies are needed to confirm the efficacy of these concepts.
